# Factors associated with unfavorable outcomes in older patients with traumatic brain injury: analysis from the *“All of Us”* research program

**DOI:** 10.3389/fneur.2024.1452995

**Published:** 2024-11-19

**Authors:** Zhangying Chen, Zihao Wang, Alexios-Fotios A. Mentis, Anne M. Stey, Steven J. Schwulst

**Affiliations:** ^1^Department of Surgery, Division of Trauma and Critical Care, Northwestern University Feinberg School of Medicine, Chicago, IL, United States; ^2^Driskill Graduate Program in Life Sciences, Northwestern University Feinberg School of Medicine, Chicago, IL, United States; ^3^Department of Statistics and Actuarial Science, University of Waterloo, Waterloo, ON, Canada; ^4^Medical School, National and Kapodistrian University of Athens, Athens, Greece; ^5^BGI Genomics, BGI-Shenzhen, Shenzhen, Guangdong, China

**Keywords:** epidemiology, traumatic brain injury, older adults, aging, all of us, prognostic tool development, disease outcomes

## Abstract

Traumatic brain injury (TBI) afflicts approximately 70 million people worldwide annually, with patients aged 65 years and older accounting for an increasing proportion of TBI patients. Older patients also experience increased morbidity and mortality post-TBI compared to their younger counterparts. Nevertheless, clinical trials often exclude older TBI patients, and age-specific TBI treatment is lacking. We hypothesized that the *APOE* genotype and age-associated comorbidities, such as heart disease, are associated with unfavorable outcomes following TBI in older patients. We utilized a dataset from the *“All of Us research”* (AoU) to study this vulnerable population post-TBI. Launched by the National Institutes of Health (NIH), AoU is a nationwide prospective cohort study aiming to enroll 1 million or more individuals by emphasizing traditionally underrepresented populations in the United States. We defined patients diagnosed with post-concussion syndromes (PCS) as those with unfavorable TBI outcomes, and we also assessed the associations between PCS observed in older patients and different comorbidities variables/APOE genotypes via multiple logistic regression models. Consequently, APOE ε4 allele was strongly associated with PCS in patients aged 65 and older. Our findings provide direct evidence for developing better predictive tools and potentially improving the clinical guidance and management of older adults with TBI.

## 1 Introduction

Trauma is a leading cause of death and disability in the United States, with traumatic brain injury (TBI) contributing to over one-third of all these trauma-related deaths (1). Adults aged 65 years and above account for over 40% of all TBI-related hospitalizations and for up to 50% of TBI-related deaths (2). Severe TBI, as measured clinically via the Glasgow Coma Scale (GCS) and/or radiographically via computed tomography (CT), in older adults is associated with a high mortality rate ranging from 50 to 80% (3–5). Most older adults who survive mild or severe TBI experience increased long-term morbidity, including TBI-related dementia, as well as slower overall recovery trajectories compared to younger adults (5). Nevertheless, a subset of older adults with TBI, including those with severe TBI, may recover well, suggesting that chronological age and TBI severity are not adequate prognostic markers on their own (6). To date, the best-known genetic risk factor for poor outcomes after TBI in adults is the ε4 allele of the apolipoprotein E (APOE) gene. This well-known gene influences the risk of neurodegenerative disorders, such as (AD) disease (7). On the contrary, the ε2 allele may provide some protection against the disease (7). Besides, the human brain-derived neurotrophic factor (BDNF) gene is another common gene important for neuroplasticity. The transition from valine to methionine substitution at codon 66 (Val66Met) of the BDNF gene can result in less active nerve growth factor and mediate alterations in gray and white matter (8, 9). The tumor protein p53 (TP53) gene is essential for DNA repair and apoptosis with one of its polymorphisms proline to arginine substitution at codon 72 (Pro72Arg) particularly driving inflammation and neuroinflammation (10, 11). Last but not least, the valine to methionine replacement at codon 158 (Val158Met) polymorphism in the catechol-O-methyltransferase (COMT) gene has been linked stress leading to diseases such as depression and anxiety in adults (12, 13). However, testing the effects of these genetic variants on TBI outcomes has proven challenging during clinical practice because the analysis requires laborious processes that might not be completed within the critical period of hospitalization.

In 2019, there were an estimated 54.1 million people aged 65 and older in the United States, and the population of older adults has been increasing consistently over the last decades (perhaps with a minor exception in the post-COVID-19 period). This demographic shift will likely lead to an additionally increasing proportion of older patients who experience TBI. Nonetheless, despite the large and growing number of older adults with incident TBI, there are no evidence-based guidelines for disease outcome prediction and management in older patients with TBI, thus leaving a major knowledge as well as *“practice gap”* (14). In addition, not all TBI studies include older adults, who represent a vulnerable population often excluded or overlooked in clinical trials of TBI (15).

The *“All of Us”* (AoU) research program launched by the National Institutes of Health (NIH) is a nationwide prospective cohort study that aims to enroll 1 million or more individuals in the United States. The program emphasizes diverse enrollment, including recruitment of communities that have been traditionally underrepresented in biomedical research; the latter represents one of the most significant advantages of working with this data source. A rich range of data from surveys, electronic health records (EHRs), physical measurements, wearables, biospecimens, and genomic data are available through AoU (16). Hence, in this case, the AoU dataset provides a unique opportunity to comprehensively evaluate the association of various risk factors with TBI outcomes in older adults.

This study aimed to examine the associations between different predictors and unfavorable TBI outcomes. We herein defined patients with unfavorable TBI outcomes as those diagnosed with post-concussion syndrome (PCS). PCS describes the constellation of symptoms persistent over time after TBI, which can be physical, cognitive, behavioral, or emotional, and includes but is not limited to headaches, dizziness, irritability, depression, anxiety, memory problems, concentration problems, and thinking problems (17). Several studies have conducted analyses to determine prognostic factors associated with PCS, with older age being a top strong predictor for long-term, persistent syndromes, such as disability, after TBI (18). Nevertheless, whether these conditions have similar prognostic values in PCS among older versus young adults with TBI has yet to be determined. We hypothesized that the *APOE* allelic variance and certain age-associated comorbidities, such as heart disease, are associated with unfavorable outcomes following TBI in older patients.

The US population is aging at an unprecedented rate, with the projected number of Americans aged 65 years and older being greater than 50% by 2050 (19). Hence, TBI in older adults is a growing public health concern, and our findings contribute to the public health relevance by deepening our understanding of risk factors associated with older TBI patients. Moreover, the findings provide direct evidence for developing better predictive tools and improving the guidance and management of older adults with TBI.

## 2 Materials and methods

### 2.1 AoU participant consent and IRB review

Informed consent was obtained from all AoU participants. Details on informed consent are available.^1^ AoU inclusion criteria include being an adult 18 and older, having the legal authority and decisional capacity to consent, and currently residing in the US or a territory of the US. The work described here was reviewed and overseen by the program’s Science Committee and was confirmed as meeting the criteria for human subject research by the AoU Institutional Review Board. All the results reported comply with the *“All of Us”* Data and Statistics Dissemination. Results reported here comply with the AoU Data and Statistics Dissemination Policy, disallowing disclosure of group counts under 20.

### 2.2 TBI outcome measure

The primary outcomes were incident diagnoses of TBI and PCS (as a surrogate of unfavorable TBI outcomes) coded in the EHR. The EHR data from participating sites were mapped and harmonized using the open community data standard Observational Medical Outcomes Partnership (OMOP) Common Data Model (20). Subsequently, unfavorable TBI outcomes, i.e., PCS versus normal TBI outcomes, were ascertained based on objective measures of functional outcomes months or years after TBI provided in EHRs. These objective measures include the global Glasgow Outcome Scale (GOS) or its extended version (GOSE) that summarizes the overall effects of TBI on function, independence, and participation (16).

### 2.3 Cohort construction

**Figure 1:**
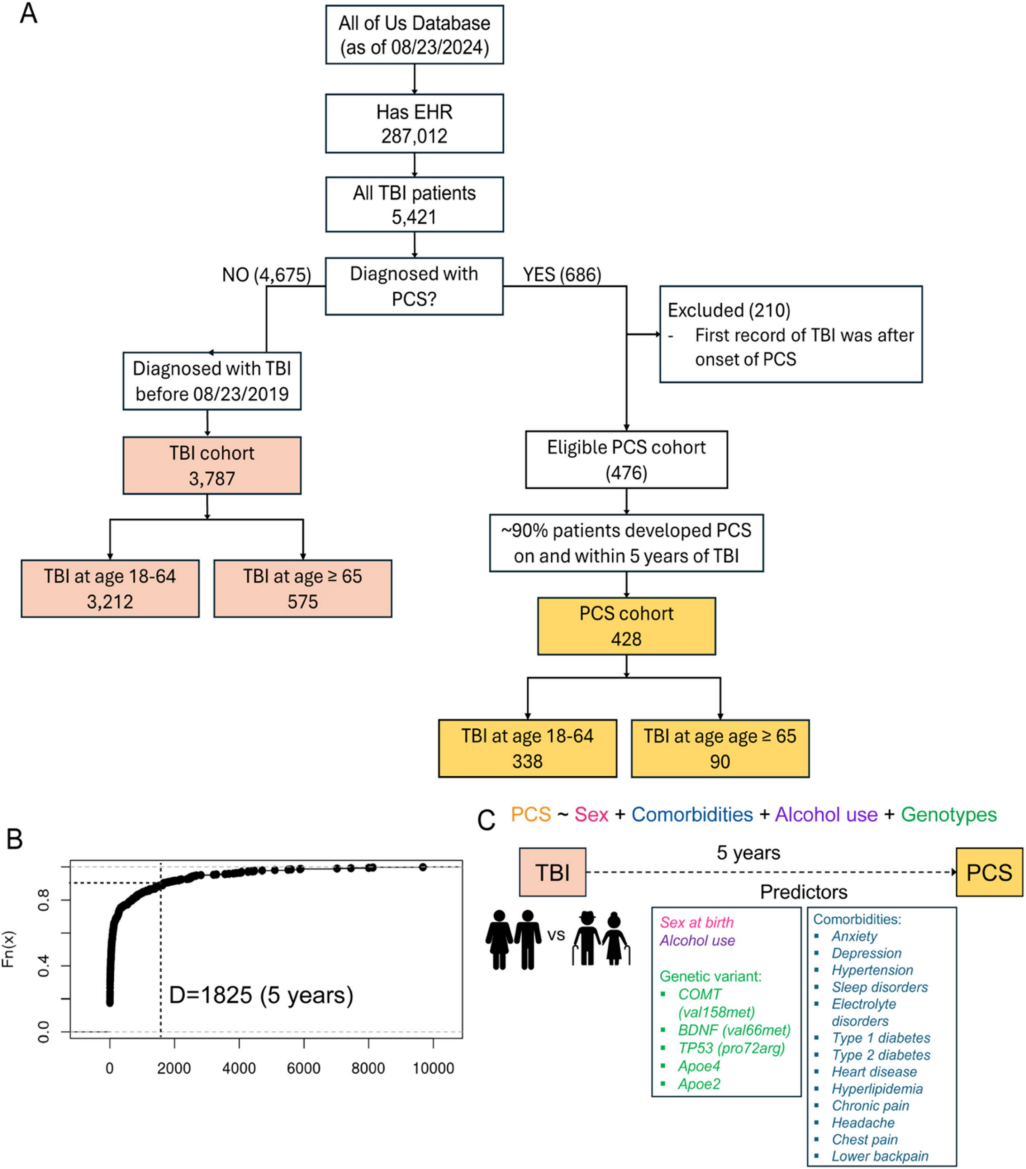
(A) Flowchart of study participant selection. EHR, Electronic health records. TBI, Traumatic brain injury. PCS, Post-concussion syndrome. (B) Empirical CDF plot displaying the time difference between the occurrence of PCS and TBI from the lowest to highest against their percentiles. Dotted lines specifying the cutoff of ~90% of PCS patients who developed PCS within 5 years after the occurrence of TBI. (C) proposed model showing various predictors and PCS as the outcome.

Participants aged 18 years and above were enrolled after providing informed consent at clinics and regional medical centers comprising the AoU research program network. For this study, we used the Registered Tier Dataset version 7 (*“C2022Q4R9 Curated Data Repository”*) available on the AoU research workbench,^2^ a cloud-based platform where registered researchers can access and analyze data. To select participants of interest, we used the Cohort Builder tool,^3^ following the AoU research workbench instruction, to include 287,012 participates with available EHR data at the time of dataset creation. Among those with EHR data, we identified a cohort of 5,421 patients with traumatic brain injury (TBI) by TBI and related SNOMED codes 127,295,002, 110,030,002, 127,302,008, 127,298,000, 127,299,008, 450,569,000, 127,301,001, 127,300,000, and 429,656,004. Next, in that cohort of TBI patients, we identified 686 patients who were diagnosed with post-concussion syndrome (PCS) by SNOMED code 40425004. PCS was our surrogate for unfavorable TBI outcomes. We kept unique patients and excluded invalid patients who had onset of PCS before their first diagnosis of TBI. By displaying the time difference between the onset of PCS and the occurrence of TBI, we found that 90% of PCS patients developed PCS within 1825 days (5 years) after the initial occurrence of TBI. Therefore, we selected 1825 days as our cutoff for how long we want to monitor our PCS/TBI cohort (Figure 1B). For the PCS cohort, we utilized a “temporal” feature, also available in the Cohort Builder, that allowed us to select participants with EHR records of PCS that happened on or 1825 days within a clinical occurrence of TBI. Based on our cutoff, we assumed those with TBI after 08/23/2019 (1825 days before the analysis) might still need more time to develop PCS if they have not yet. Hence, for the TBI cohort, we only included patients diagnosed with TBI before 08/23/2019. Consequently, we identified a cohort of 3,787 TBI patients and a cohort of 428 PCS. We further divided these two cohorts by the age of having TBI. In the TBI cohort, we had 3,212 patients who had TBI between 18 and 64 and 575 patients aged 65 and older. In the PCS cohort, we had 338 patients who had TBI between 18 and 64 and 90 patients aged 65 and older (Figure 1A). Participant demographics (age, self-reported gender, sex at birth, and race/ethnicity) were derived from survey data completed at the time of enrollment.

### 2.4 Comorbidity data

Based on curated literature, comorbidities in older patients with TBI range from psychiatric comorbidities (anxiety and depression), sleep disorders, chronic pain, hypertension, heart disease, and diabetes (Type 1 and Type 2) (12–21). Other comorbidities derived from TBI include electrolyte disorders, hyperlipidemia, and chronic pain at different locations (head, chest, and lower back) (22–24). EHR results were utilized to identify comorbidities following TBI using SNOMED codes. We utilized the “temporal” feature in the Cohort Builder to select participants with EHR records indicating various comorbidities that happened on or 1825 days within a clinical occurrence of TBI.

### 2.5 Genetic variants

Based on curated literature, genetic variants of interest include COMT (val158met), BDNF (val66met), TP53 (pro72arg), APOE ε2 and ε4 alleles (7–13). We accessed genomic data under the short read whole genome sequencing (srWGS) data through the Controlled Tier dataset version 7. We used the Cohort Builder tool to extract all the carriers of genotypes of interest by rsID number: rs429358 for APOE ε4 allele, rs7412 for APOE ε2 allele, rs6265 for BDNF (Val66Met), rs1042522 for TP53 (Pro72Arg) and rs4680 for COMT (val158met).

### 2.6 Alcohol use

We assessed alcohol use data via the lifestyle survey through the Registered Tier dataset version 7. We queried the data to the specific question in which AoU participants estimated the number of drinks they typically have every day at the time of enrollment (question ID: 1586207).

### 2.7 Statistical analysis and data visualization

Of the data containing all the predictors, we identified each unique participant ID and annotated the data with our cohorts accordingly. All analyses for the present study were performed entirely on AoU workbench using RStudio version 4.4.0 (see Footnote 2). To study risk factors associated with PCS, associations between the case cohort PCS (outcome) and different comorbidity variables, genetic variants, sex at birth, and alcohol use (predictors) were tested via multiple logistic regression models. Separate analyses were performed for aged 18–64 versus 65 years and older. The model was used to calculate odds ratios and 95% confidence intervals for each predictor. The R package ‘dominanceanalysis’ version 2.1.0 was used to rank the relative importance of predictors in the multivariable logistic regression model (25).

### 2.8 Data and code availability

The datasets used and analyzed in the present study are available to US based researchers via the researcher workbench (see Footnote 2). All code can be found.^4^

## 3 Results

Between May 2018 and Aug 2024, more than 821,000 participants enrolled in the AoU program, and 287,012 had EHR data available at the time of dataset creation. Among those with EHR data, we identified a cohort of 5,421 patients with traumatic brain injury (TBI) by TBI and related SNOMED codes 127,295,002, 110,030,002, 127,302,008, 127,298,000, 127,299,008, 450,569,000, 127,301,001, 127,300,000, and 429,656,004. Next, in that cohort of TBI patients, we identified 686 patients who were diagnosed with post-concussion syndrome (PCS) by SNOMED code 40425004. By displaying the time difference between the onset of PCS and the occurrence of TBI, we found that 90% of the patients developed PCS within 1825 days (5 years) of the initial occurrence of TBI. Therefore, we assigned 1825 days as our cutoff for downstream analysis, and we only included patients diagnosed with TBI before 08/23/2019 (1825 days before the time of analysis). Consequently, we identified a cohort of 3,787 TBI patients and a cohort of 428 PCS. We further divided these two cohorts by the age of having TBI. In the TBI cohort, we had 3,212 patients who had TBI between 18 and 64 and 575 patients aged 65 and older. In the PCS cohort, we had 338 patients who had TBI between 18 and 64 and 90 patients aged 65 and older (Figure 1A).

**Figure 2:**
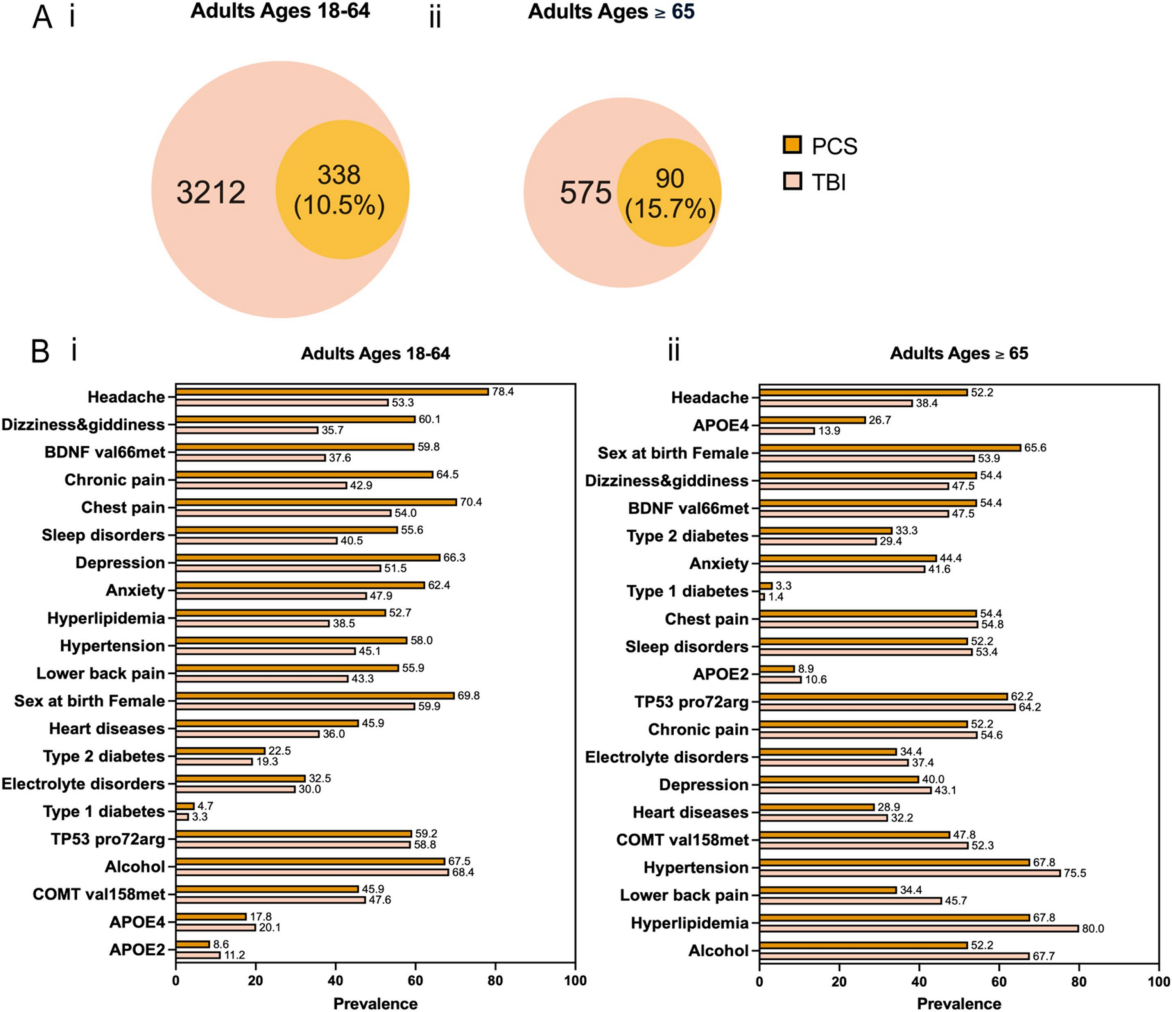
(A) Venn diagram for co-occurrence of TBI and PCS across two age groups. (B) Prevalence of the 21 predictors for PCS versus TBI across two age groups. The predictors are arranged in descending order based on the magnitude of their difference in prevalence between PCS and TBI.

Characteristics of study participants by gender and race/ethnicity for each cohort and age group are presented in Table 1. We observed more female patients across two cohorts and different age groups: 59.9% in patients with TBI aged 18–64, 53.9% in patients with TBI aged 65 and older, 69.8% in patients with PCS aged 18–64, and 65.6% in patients with PCS aged 65 and older. This disproportion can be explained by more females than males inherent to AoU datasets (59% vs. 39%). Next, we divided each cohort and age group by TBI-related clinical characteristics based on the Observational Medical Outcomes Partnership (OMOP) standard concept names (Table 2). Concussion was the most common form of TBI across two cohorts and age groups: 71.4% in patients with TBI aged 18–64, 67.1% in patients with TBI aged 65 and older, 77.6% in patients with PCS aged 18–64, and 71.2% in patients with PCS aged 65 and older. As expected, aged patients have more likeliness to have unfavorable TBI outcomes indicated by PCS than their younger counterparts (15.7% vs. 10.5%; Figures 2Ai,ii).

**Table 1:** Characteristics of TBI patients with or without PCS development by age, sex at birth and race.

PCS development	Age at first TBI	Sex at birth	White	Black	Asian	≥ 1 pop.	Total
No	18–64	Female	1,401 (43.6%)	405 (12.6%)	44 (1.4%)	60 (1.9%)	1925 (59.9%)
		Male	866 (27.0%)	355 (11.1%)	24 (0.7%)	33 (1.0%)	1,287 (40.1%)
	≥65	Female	274 (47.7%)	28 (4.9%)	<20	<20	310 (53.9%)
		Male	242 (42.1%)	<20	<20	<20	265 (46.1%)
Yes	18–64	Female	187 (55.3%)	30 (12.6%)	<20	<20	236 (69.8%)
		Male	86 (25.4%)	<20	<20	<20	102 (30.1%)
	≥65	Female	52 (57.8%)	<20	<20	<20	59 (65.6%)
		Male	24 (26.7%)	<20	<20	<20	31 (34.4%)

Values under the reporting minimum are represented as ‘< 20’. ≥ 1 pop.: more than one population.

**Table 2:** Characteristics of TBI patients with or without PCS development by the Observational Medical Outcomes Partnership (OMOP) standard concept names.

Standard concept names	Concept Id	TBI at 18–64 (3212)	TBI at **≥**65 (575)	TBI to PCS at 18–64 (338)	TBI to PCS at **≥**65 (90)
Concussion					
Concussion, no loss of consciousness	378,001	945 (29.4%)	167 (29.0%)	120 (35.5%)	41 (45.6%)
Concussion, loss of consciousness	375,671, 4,019,263	736 (22.9%)	119 (20.7%)	59 (17.5%)	23 (25.6%)
Concussion, unspecified	4,001,336	612 (19.1%)	100 (17.4%)	83 (24.6%)	<20
TBI					
TBI, no open intracranial wound	4,234,112	402 (12.5%)	37 (6.4%)	24 (7.1%)	<20
TBI, loss of consciousness	4,133,017, 4,133,018, 4,132,083	69 (2.1%)	20 (3.5%)	<20	<20
Post-traumatic cerebral infarction	443,454, 4,110,192	85 (2.6%)	27 (4.7%)	<20	<20
Intracranial hemorrhage, loss of consciousness	444,197	88 (2.7%)	<20	<20	<20
TBI, unspecified	4,132,546	40 (1.2%)	<20	<20	<20
TBI, no loss of consciousness	4,133,715	32 (1.0%)	<20	<20	<20
Subdural hemorrhage, no open intracranial wound, no loss of consciousness	438,595	23 (0.7%)	<20	<20	<20

Values under the reporting minimum are shown as ‘< 20’.

To study potential factors associated with PCS, we curated a list of predictors comprising sex at birth, alcohol use, genetic variants, and comorbidities (Figure 1C). Genetic risk factors can influence the likelihood of developing PCS after TBI. They might have a differential impact on PCS development in older patients than younger ones. Based on curated literature, we selected the apolipoprotein E (APOE) ε4 allele and ε2 allele, the valine to methionine substitution at codon 66 (Vasl66Met) single nucleotide polymorphism (SNP) in the brain-derived neurotrophic factor (BDNF) gene, the proline to arginine substitution at codon 72 (Pro72Arg) SNP in the tumor protein p53 (TP53) gene, and the valine to methionine replacement at codon 158 (Val158Met) SNP in the catechol-O-methyltransferase (COMT) gene (citations). Similarly, comorbidities after TBI can complicate recovery and exacerbate symptoms, and they might differentially affect older than younger patients. We included medically diagnosed comorbidities as listed below: anxiety, depression, hypertension, sleep disorders, electrolyte disorders, Type 1 diabetes, Type 2 diabetes, heart disease, hyperlipidemia, and chronic pain (head, chest, and lower back). Besides, alcohol intake and sex at birth were two additional predictors. The prevalence of different predictors in the TBI and PCS cohorts divided by age groups is shown in Figure 2B. In patients aged 18–64, the most significant difference between PCS and TBI was noted in the prevalence of headache (78.4% vs. 53.3%) followed by dizziness and giddiness (60.1% vs. 35.7%) and BDNF (Val66Met; 59.8% vs. 37.6%; Figure 2Bi). Likewise, older PCS patients had a higher prevalence of headache than TBI patients (52.2% vs. 38.4%). Besides, older PCS patients had a noticeably increased prevalence of APOE4 allele than older TBI patients (26.7% vs. 13.9%; Figure 2Bii).

Subsequently, participants without information pertaining to sex at birth were removed for downstream analyses. We performed a correlation analysis to test the co-existence of any two variables. As a result, PCS and dementia were highly related, followed by BDNF (Val66Met) and dizziness and giddiness, and COMT (Val158Met) and TP53 (Pro72Arg) indicated by correlation values of 0.83, 0.74, and 0.56, respectively. Therefore, we proceed with removing dementia, dizziness giddiness, and TP53 (Pro72Arg) for multivariable logistic regression analysis.

**Figure 3:**
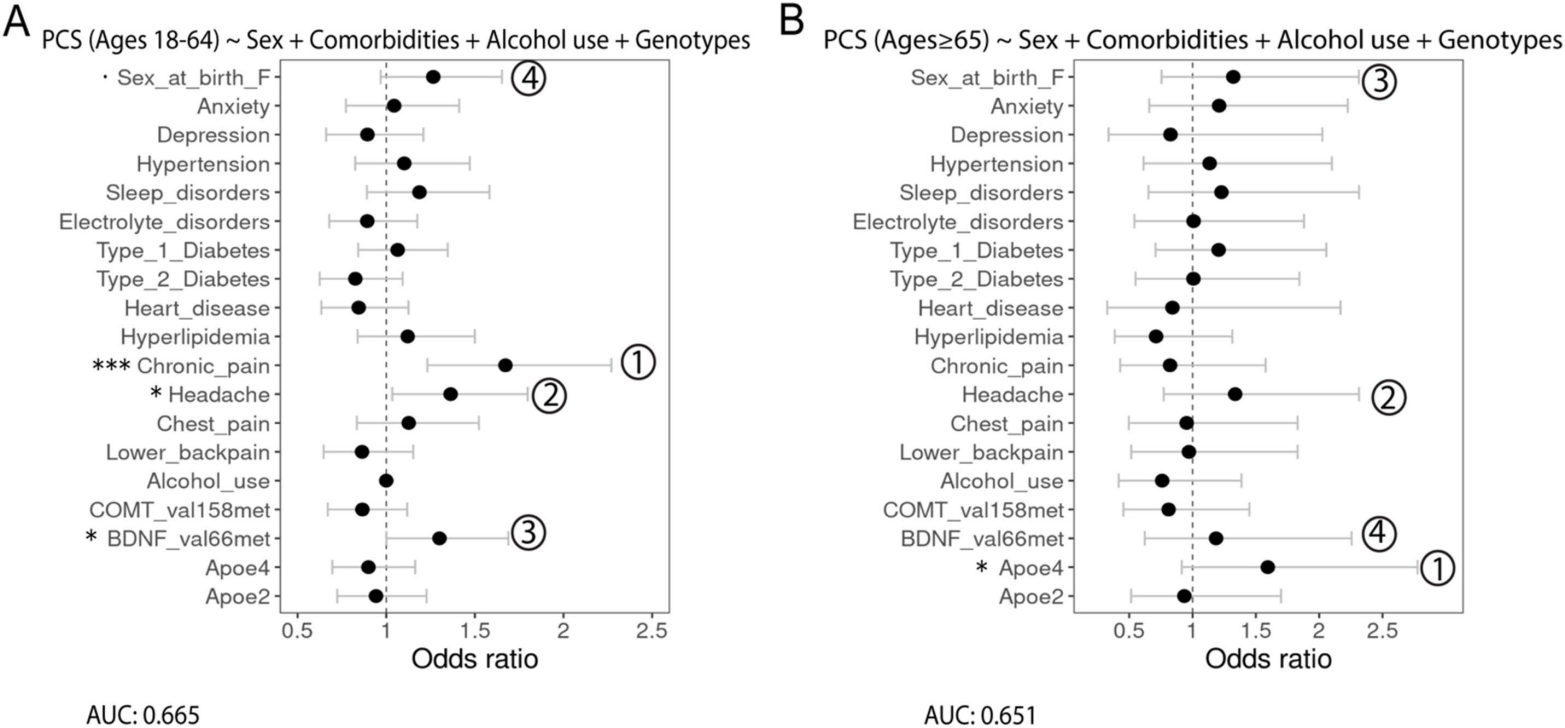
Forest plots showing the association of different predictors with PCS outcomes in adults aged (A) 18–64 years and (B) 65 years and older with the AUC score being reported under the formulation of the model. Numbers show the relative importance of the predictors obtained using dominance analysis. Significance codes: 0 ‘***’ 0.001 ‘**’ 0.01 ‘*’ 0.05 ‘.’ 0.1.

First, multivariable logistic regression assessing the associations between PCS, as the outcome, and gender and race/ethnicity, as the predictors, indicated a significant association with PCS and sex at birth (female), and race/ethnicity was not significantly associated with PCS (data not shown). Next, we modeled the associations between PCS and sex at birth, alcohol use, comorbidities, and genetic variants, with dominance analysis used to compare the relative importance of the predictor for both age groups (Figure 3). Among patients aged 18–64, chronic pain (OR = 1.6, 95% CI 1.05–1.28, *p* = ***) was the leading risk factor most strongly associated with PCS in addition to headache (OR = 1.36, 95% CI 1.03–1.80, p = *) and BDNF (Val66Met; OR = 1.30, 95% CI 1.00–1.69, *p* = *). Being a female at birth is slightly associated with PCS (OR = 1.27, 95% CI 0.97–1.65, *p* = .). Dominance analysis further showed that these factors were the top predictors of PCS (Figure 3A). Among older patients, the APOE4 allele was statistically associated with PCS (OR = 1.59, 95% CI 0.92–2.78, *p* = *). It was also the most relatively important factor for PCS based on dominance analysis, followed by headache, being a female at birth, and BDNF (Val66Met; Figure 3B).

## 4 Discussion

In this analysis of outcomes following TBI among older patients in the AoU research program, APOE allele was significantly associated with unfavorable TBI outcomes indicated by PCS. To study the association between APOE alleles and TBI outcomes, animal experimental models have been extensively used in lieu of obtaining human samples from clinical studies. The APOE protein is known to maintain synaptic integrity, promote neural recovery and repair, and to regulate inflammatory response after brain injury (26–28). The human APOE gene exists in three polymorphic alleles – ε2, ε3, and ε4 – which have a population frequency of 8.4, 77.9, and 13.7%, respectively (29). Of note, the APOE ε4 allele, compared to APOE ε2 allele, has a neurotoxic effect causing decreased neural repair mechanisms and greater vulnerability to neurodegeneration (30, 31). Our findings further show that APOE ε4 allele status is differentially associated with unfavorable TBI outcomes, including a higher risk of PCS in the older patients but not the younger counterparts. The more pronounced effect of APOE ε4 allele in older patients than their younger counterparts might be due to the increased susceptibility to ε4 allele, declined robust brain plasticity, and cognitive reserve (32). On the other hand, the risk factors associated with unfavorable TBI outcomes indicated by PCS in the younger patients are chronic pain, headache, and BDNF (Val66Met) SNP. Chronic pain following TBI is a common comorbidity varied by locations (33). Nevertheless, chronic pain, along with headache, was less prevalent in older patients. Its effect was also less pronounced in older patients than younger ones. Hence, we speculated an age-associated disruption in pain perception, as indicated by reduced sensitivity for pain of low-medium intensity in older adults (34). In summary, TBI is a chronic disease process that has long-lasting effects on patients. Understanding different risk factors for unfavorable outcomes could guide care and assess prognosis in a more personalized approach and improve long-term disease management. Specifically, an age-associated approach is necessary. Based on our findings, the APOE4 allele is the most potent risk factor predicting PCS in older patients, along with the BDNF (Val66Met) SNP, another gene polymorphism important for neuroplasticity. Hence, genetic testing will help identify potential risks associated with long-term unfavorable TBI outcomes.

This analysis has major strengths, such as the large cohort size and racial/ethnic diversity of the participants. Datasets from the *“AoU research program”* allow merging EHR from multiple centers to become feasible. Assessing TBI outcomes is difficult in older adults as they have multiple age-associated conditions such as heart disease, hypertension, and so on, so it may be impossible to isolate the effect of TBI on these conditions. Our analysis has addressed this challenge by systematically measuring these conditions rather than simply excluding them. Moreover, incorporating a wearable device and other at-home visits or telehealth follow-up options, some older adults with previous physical limitations due to TBI and other comorbidities can continuously participate in the program. With that, the AoU research program and similar programs will eventually provide more longitudinal data to help better understand the recovery trajectory of patients with TBI, along with many other diseases, so that we can repeat this analysis from time to time or involve machine learning to construct accurate prognostic models. This study also has several limitations. First, there are limitations of using EHR data for TBI outcomes with a potential lack of specificity of diagnostic codes and a difficulty in distinguishing repeated TBI from the initial TBI insult. Second, we used PCS as a surrogate endpoint for unfavorable TBI outcomes, an approach which can increase the ambiguity of the results due to the complexity of TBI. A more comprehensive examination of different sequelae and ensuing secondary injury post-TBI using the AoU research datasets is necessary. Lastly, further analysis should incorporate medications such as anticoagulants in the statistical model and also consider adjustment for multiple comparisons.

Longitudinal cohort studies, including the AoU research program, have significantly advanced our understanding of human health by establishing direct correlations between disease outcomes and various risk factors. While multiple regression analyses have identified distinct age-associated risk factors predicting unfavorable long-term TBI outcomes, insights from animal models are equally critical by elucidating molecular and cellular alterations that occur in aged brains post-TBI. Recent studies have concentrated on deciphering varying age-related responses to brain injury, deepening our understanding of the disparate CNS response in young versus aged subjects following TBI and pinpointing potential therapeutic targets for age-specific TBI treatments (35–52).

Post-TBI, age-related responses involve complex, multifactorial biological processes in the tri-layered meninges, blood–brain barrier (BBB), and brain parenchyma, mediated by glial cells such as microglia and astrocytes, as well as cytokines and infiltrating immune cells (35–52). By assessing these responses, preclinical animal studies have highlighted promising age-specific TBI treatments. For instance, aging increases BBB permeability and TBI activates matrix metalloproteinase proteins (MMPs) that further contribute to BBB breakdown in aged mouse brains compared to younger ones (36–39). Consequently, targeting the BBB with pharmacological MMP inhibitors could offer a promising avenue for treating older adults post-TBI (40, 41).

Immediately post-TBI, microglia and astrocytes trigger immune responses by releasing cytokines and chemokines and recruiting peripheral immune cells (42–44). While the initial activation of microglia and astrocytes helps resolve injury and promote tissue repair and remodeling, prolonged activation can amplify neuroinflammation, particularly in aged individuals whose microglia and astrocytes are primed for chronic activation (44–48). Aging also leads to an exaggerated cytokine response and disproportionate CD8+ T cell infiltration long-term post-TBI, contributing to unresolved neuroinflammation (49). Therapies inhibiting lymphocyte migration into the CNS may mitigate these age-related TBI effects. For instance, Natalizumab, an approved drug for MS, has been found to improve survival and neurocognitive functions in aged mice post-TBI by reducing CD8+ T cell infiltration and pro-inflammatory cytokines (49).

Collectively, age-associated alterations in the transcriptional and cellular landscape can prolong harmful post-injury responses, causing further neuronal loss and a feedforward cycle of neuroinflammation and neurodegeneration (44), thereby leading to aggravated TBI outcomes commonly seen in aged individuals. Promising treatments in younger individuals have demonstrated diminished preclinical efficacy in older adults and failed in clinical trials, underscoring the critical need to understand these age-related alterations (50–52). Findings from the present study advance the search for age-specific TBI treatment. The strong association between the APOE ε4 allele and unfavorable outcomes in older patients spotlights the potential for using amyloid-removing antibodies or other therapies targeting APOE4. Together, findings from the present study provide direct evidence for developing better predictive tools and potentially improving the guidance and management of older adults with TBI.

## Data Availability

The datasets presented in this article are not readily available due to the All of Us privacy policy regarding patient anonymity. Requests to access the datasets should be directed to the corresponding author.

## Footnotes

^1^https://allofus.nih.gov/sites/default/files/aou_operational_protocol_v1.7_mar_2018.pdf

^2^https://www.researchallofus.org/data-tools/workbench/

^3^https://support.researchallofus.org/hc/en-us/articles/360039585591-Selecting-participants-using-the-Cohort-Builder-tool

^4^https://github.com/Jennieecc/allofus_tbi
